# The Metropolitan Asylums Board Report for 1920-21

**Published:** 1921-09-17

**Authors:** 


### AN EXCELLENT RECORD.

### The Metropolitan Asylums Board Report for 1920-21.

The Metropolitan Asylums Board was constituted in 1867, "for the reception and relief of the classes of poor persons chargeable to some union or parish in the said districts respectively who may be infected with or suffering from fever or the disease of smallpox, or may be insane." But during the last half-century the sphere of its activities has constantly widened, and now we find under its administration fourteen hospitals for infectious diseases with arrangements for dealing with plague and cholera; bacteriological establishment and laboratories; eight sanatoria and hospitals for tuberculous patients (under the National Insurance Act, 1911-1913); two institutions for women and girls suffering from venereal disease; a hospital for cases of ophthalmia neonatorum; three hospitals for imbeciles; an infirmary for aged patients; two training colonies for improvable imbeciles and feeble-minded; two colonies for sane epileptics; three institutions for sick children; an institution for ringworm and other skin diseases; an ophthalmia school; two training-ships for boys; six casual wards for homeless poor; night office for homeless poor; seven ambulance stations; three riverside wharves, with motor ambulances and ambulance steamers; central stores for the collection and distribution of goods to the various institutions. Altogether there are fifty residential institutions under the care of the Asylums Board, and these have, on the average, a resident population of about 25,000—16,000 patients and 9,000 staff. The Board itself is composed of seventy-three members, fifty-five being elected by the Metropolitan Boards of Guardians and eighteen nominated by the Ministry of Health.

The work of the Board, as summarised above, evidently covers a very large field, and the report for the year 1920-21 shows that in every department work of great value is being done.

The epidemics of scarlet fever and diphtheria in the latter months of 1920 naturally taxed the resources of the fever hospitals to their utmost limit. From July 3 to December 31 there were 23,179 cases of infectious diseases admitted, and on November 23 there were 8,669 patients in hospital, 1,511 more patients than had ever previously been in residence. On sixteen days more than 200 patients were admitted during the twenty-four hours. Despite such unprecedented pressure, the Board and their staff succeeded in meeting the situation, and received a letter of appreciation from the Ministry of Health.

During the year there were five small outbreaks of smallpox in London, and fifty cases were admitted by the Asylums Board.

It is satisfactory to learn that the Board and the London County Council have succeeded in obtaining beds in hospitals and sanatoria sufficient to meet the estimated requirements of tuberculous adults and children. In addition, arrangements have been made for education and after-care of tuberculous children, and for some training of tuberculous adults in such manual occupations as wood-work, leather-work, wicker-work, and brush-making.

There were 875 new cases admitted to the Board's mental hospitals during the year, and on December 31, 1920, there were 5,539 cases in residence. The admissions during the year to the Board's hospitals, schools, and homes for sick children amounted to 2,106, and at the close of the year there were 1,429 children in residence.

In the casual wards 15,478 casual poor were received—a daily average of 136. The report gives interesting and instructive comparative figures for the years 1912 to 1921 inclusive. It shows a remarkable decrease in the casual poor during the years of war. In January 1912 the casual wards received more than fifteen times the number of casual poor received in January 1919, and in September 1912 fifteen times the number received in September 1918. Now, unfortunately, the figures are going up again, and in December 1920 there were almost five times as many casual inmates as in 1918. The Casual Wards Committee is not content with merely receiving the casual poor; it supplies magazines, endeavours to find work and "to improve the condition, and in association with voluntary agencies to uplift and restore every possible case coming under the Board's care." The Board's night office, working in conjunction with the Homeless Poor Committee, has dealt during the year with 7,635 cases of homeless poor.

The report contains some very interesting appendices. Appendix C, by the Very Rev. Canon Sprankling, treats of the work of the Metropolitan Asylums Board for the mentally defective, and is especially interesting in view of the attacks lately made on mental institutions. While it is admitted that in some cases the infirmities of the mentally defective children are "so many and so overwhelming that it is hardly possible to give them any instruction or training," yet where possible children are trained and "improved," and at Fountain Mental Hospital especially excellent work is being done under a teacher-nurse. "She started by teaching them [the mentally defective children] how to play, and gradually advanced them through elementary kindergarten work, musical drill, and singing, with action-song and dancing, to doll-making, mat-making, and the dressing of dolls. . . . No one who sees the children as they are on admission can fail to be struck with the remarkable change there is in them, in most cases after a comparatively short period. The patience and the care which must be lavished on these afflicted atoms before they reach the standard of education displayed can hardly be appreciated, except by those who watch the work of the institution constantly.

Those directing the work must be whole-heartedly enthusiastic to have achieved such results as are visible here, where apparently the most hopeless of the mentally deficient have been gathered together."

Appendix D, by T. B. Layton, D.S.O., M.S.Lond., F.R.C.S.Eng., is a valuable report, dealing mainly with the symptoms and treatment of ear disease in scarlet fever. Mr. Layton is not an advocate of syringing the throat, and believes that the best results are obtained by early paracentesis. "In the vast majority of cases," he affirms, "if this operation is performed at the right time the inflammation of the ear will clear up, and chronic ear discharge will not ensue."

There are other appendices on the laboratory work of the Board by Sir G. Sims Woodhead, M.A., M.D., LL.D., and G. E. Cartwright Wood, M.D., B.Sc.; on the treatment of ophthalmia by Mr. L. J. Pisani, F.R.C.S., and Mr. E. Treacher Collins, F.R.C.S.; and on the treatment of skin diseases by Sir James Galloway, K.B.E., C.B., M.D., F.R.C.P.

The report includes some seventy pages of statistical tables.

The work done by the Metropolitan Asylums Board seems so excellent and efficient that the proposed transference of its duties to the London County Council would seem to be a step of doubtful expediency, even if members of the Board be co-opted on the new Council Committee. The objections to the present system are mainly overlapping of functions and some poor-rate anomalies; but these seem hardly sufficient to justify interference with machinery which has otherwise worked so well. Centralisation may prevent overlapping, but decentralisation makes for efficiency, and fifty-five years' practical administrative experience is an asset not easily replaced.

